# India and Indian cardiothoracic surgery: an insider brief 40-year perspective

**DOI:** 10.1007/s12055-025-02009-3

**Published:** 2025-07-04

**Authors:** Carlos - Alberto Mestres

**Affiliations:** Department of Cardiothoracic Surgery, The Robert WM Frater Cardiovascular Research Centre, The University of the Free State, 9300 Nelson Mandela Drive, Bloemfontein, South Africa

**Keywords:** India, History, Cardiothoracic surgery, Indian Association of Cardiovascular-Thoracic Surgeons, *Indian Journal of Thoracic and Cardiovascular Surgery*

## Abstract

The Indian Association of Cardiovascular-Thoracic Surgeons is a mature organization that has had an unprecedented growth in the past four decades. The *Indian Journal of Thoracic and Cardiovascular Surgery* is the official publication, its visible face. It has undergone substantial changes in organization and structure and has recently been given an impact factor of 0.7. This contribution is a quick individual view of a surgeon exposed to the reality of India and Indian cardiothoracic surgery during the past four decades.

## Introduction

I first landed in India on February 4, 1985, from Barcelona, arriving at the Bombay Sahar International Airport, located to the northeast of the Royal Air Force Santacruz Station. I was due to attend a major event, the World Conference on Open Heart Surgery organized by the late KR Shetty and GB Parulkar, at the Taj Mahal Palace Hotel. President Giani Zail Singh inaugurated the conference.

For a young attending surgeon at the Hospital Clinic of the University of Barcelona in Spain, it was exciting having two abstracts for oral presentation. The list of foreign luminaries was endless. This conference opened my eyes regarding India and Indian cardiothoracic surgery. After enjoying the conference and strolling a bit in town, I flew back home on February 11, 1985. This was an inspirational experience personally and professionally. It was the beginning of a lifelong relationship with India, Indian cardiothoracic surgery, and Indian cardiothoracic surgeons.

This historical vignette cum narrative review aims at summarizing 40 years of individual exposure to the country and Indian cardiothoracic surgeons, to understand how these four decades crafted the current Indian cardiothoracic scenario.

## What in 1985 and today?

I arrived before the liberalization and economic reforms of 1991. In 1985, the population of India was 780 million, its gross domestic product (GDP) 232.5 billion in US dollars, and its purchasing power parity (PPP) 4.667 [1]. To make it easy, when I first came, there was only Campa-Cola, and the big tech companies were dreaming of entering India. There were no cell phones.

India has risen to become the world’s fourth-largest economy by nominal GDP and the third-largest by PPP, with the GDP nearly doubling every year in the past decade. The country recovered from the pandemic, with a 13.5% GDP growth in Q1 of the Financial Year 2023, with some expected fluctuations, with a 4.27 trillion United States Dollar (USD) and a 6.2 annual percent change for 2025, according to the International Monetary Fund [2]. It is the most populous country in the world, with 1.46 billion people, overtaking China in 2022. India is now the second country in the world in cell phones (1.143 billion) with 78.1 cell phone lines per 100 citizens (https://worldpopulationreview.com/country-rankings/cell-phones-by-country).

## The Indian Association of Thoracic and Cardiovascular Surgeons

The history of the Association and cardiothoracic surgery in India is well known. I will highlight some relevant details. The cardiovascular and thoracic surgeons of India first met in Nagpur in 1954 under the umbrella of the Association of Surgeons of India. From this small 15-member gathering, first presided over by Dr. Reeve H. Betts, the Indian Association of Cardiovascular-Thoracic Surgeons (IACTS) has had a steady, unprecedented growth and development. Initially registered as the Association of Thoracic and Cardiovascular Surgeons of India (ATCVSI) in Delhi in 1982, its first independent meeting as IACTS of today was held in Madras in 1988 and I attended. Today, IACTS is a mature organization, with a solid organizational structure and a membership roster in excess of 2000 dedicated to the advancement of science and the defense of professional interests of the cardiothoracic community for the benefit of the patients.

The motto of IACTS, expressed in its renovated website, is simple: there is excellence and dedication through decades of experience, involving innovation, transformation, and evolution, a strong message delivered in a simple way. This is what IACTS has achieved over these seven decades. Today, IACTS is a worldwide recognized organization and has a growing presence in the international arena, participating in societal projects, incorporating sister organizations into domestic activities like Annual Conference of IACTS (IACTSCON), and undertaking projects of national impact like the IACTS database. Thus, IACTS endorsed the 2024 European Association of Cardio-Thoracic Surgery (EACTS)/Society of Thoracic Surgeons (STS) Guidelines of the aortic organ [3]. The American Association for Thoracic Surgery (AATS)/IACTS Cardiothoracic Postgraduate Course at the annual IACTSCON is almost a tradition. The STS/South Asian/IACTS Collaboration has recently been introduced. Just a few examples of IACTS outreach and how Indian cardiothoracic surgery is expanding.

Education is a part of IACTS, currently expressed through the Fellowship Programmes, 12 available to juniors for extended education, covering from congenital heart disease to mechanical circulatory support and 19 institutes offering them. Even in times of crisis, as during the pandemic, IACTS promptly responded and the Masterclasses, with experts from India and abroad, were a success, turning difficult times for education into useful activities for the community.

In the same line, the mid-term Cardiac and Thoracic continuous medical education (CME) and Workshop aims at sharing recent advancements in a variety of fields. The IACTS Score, running for about a decade now, a premier educational program for residents, delves in Cardiothoracic and Vascular Surgery ensuring maximum exposure to practical learning. Other courses and workshops are endorsed by IACTS. This confirms that IACTS is alive and has a bright future. Further, IACTS has pioneered off-pump coronary artery bypass (OPCAB) in the world and is at the forefront of minimally invasive cardiac surgery (MICS), foreseeable more than two decades ago [4]. Additionally, IACTS fiercely stood up to officially position itself in times of scientific disarray [5], which reinforces its stance as a solid, independent scientific organization.

Attending the Bombay conference was my first exposure to the country and the system. I was, however, privileged over the years to participate in a variety of activities across the country. One of my fondest memories was the invitation from the late KM Cherian to participate in the International Workshop “Updates in Cardiology and Cardiac Surgery” held at the Madras Medical Mission on January 11–13, 1991. I was overwhelmed to be one of the operating surgeons next to Donald Ross from London, Pantpis Sakornpant from Bangkok, Alan Kerr from Auckland, and David McGiffin then in Brisbane. My lifelong relationship with IACTS has been rewarding professionally and personally. Initially, as an Overseas Life Member, I was later inducted as an Honorary Member of IACTS in February 2011 at the Mamallapuram Annual Conference, one of the highlights of my career. I am extremely proud of it and thankful to IACTS for bestowing this honor upon me.

## The *Indian Journal of Thoracic and Cardiovascular Surgery*

The *Indian Journal of Thoracic and Cardiovascular Surgery* (IJTC) was launched in October 1982 with 31 articles, the late Solomon Victor as its first Editor-in-Chief (1982–1990). After his sudden passing [6], Amit Banerjee (1992–1996) and Jayant Kharbase (1997–1999) followed. Currently in its 41 st volume, the IJTC has undergone major organizational and structural changes. The frequency of publication and the number of volumes per year suffered significant fluctuations between 1982 and 1991; only one yearly issue was published, remembering that in 1986, 1988, and 1990 it was not published. Two yearly issues were published between 1992 and June 2000 when Arkalgud Sampath Kumar (2000–2011) took over. Four issues were consistently published thereafter, starting with volume 17 in 2001.

Supplement issues, an important part of scientific journals, focus on a given topic and experts in the field are invited to contribute. Supplement issues reinforce the role of a journal as a vehicle for knowledge transfer. The first Supplement Issue of the IJTC was published in January 2004 to celebrate the 50 th birthday of IACTS. The most prominent figures from India and abroad reviewed what had happened in these first 50 years and opinions from cardiology, anesthesia, perfusion, and physiotherapy gathered, an excellent summary of the first part of the history of IACTS. From 2018 onwards, special issues/supplements have regularly been organized to enlighten us on specific topics.

At the end of Sampath Kumar’s decade, IJTC was a journal rescued from local irrelevance and brought to a relevant international recognition [7]. In the past 15 years, Krishna Iyer (2011–2016) and OP Yadava took the responsibility to bring IJTC to even higher levels. What we have today? IJTC is a established journal with regular supplement issues. IJTC is consolidated as a monthly journal, including regular supplement issues, under a hybrid publishing model, with around 200,000 downloads registered and counting, an h5 index of 12, and a short, less than 6 weeks, interval between acceptance and publication. In 2021, IJTC was indexed on PubMed Central™, another cornerstone in international recognition. Secretary Hiremath, on behalf of IACTS and IJTC, wrote a few lines with an intense emotional charge as a reminder of the collective effort.

Finally, despite this being controversial with regard to its actual value, on Jun 8, 2023, the IJTC was assigned, for the first time, an impact factor (IF) of 0.7. This was great news as IF, likely the most widely used scient metric index, reflects the relative importance of a publication. I was particularly happy, as I was when IJTC was indexed, and I voiced it on this forum. What should follow is a steady flow of Indian contributions capturing the best Indian science.

The future is here. Our current Editor-in-Chief confirmed that IJTC is a monthly journal since January 2025, the marker of respectability, reputation, and readership attained. Last month of February, IACTS has chosen Pradeep Narayan to receive the relay in this long-distance and obstacle course race. The IJTC will grow, and the harvest will be parallel to the efforts of all those who contribute.

## Closing

In summary, India and Indian cardiothoracic surgery have made extraordinary progress in the past seven decades. As an insider myself in the broad sense, I am a privileged 40-year witness of such an unprecedented development of surgery, from rheumatic disease to the most complex congenital defects, of IACTS itself and of the IJTC, even in times of fear and uncertainty and suboptimal resources. I am proud and honored to have been accepted in this organization. It was worth it then and I have never regretted my first landing in Bombay.

## Data Availability

All that is disclosed in this contribution is publicly known.

## Declarations

No artificial intelligence has been used to write this contribution.
